# Dr. Hering’s Repertory of Tooth-Ache

**Published:** 1899-12

**Authors:** 


### HOMŒOPATHIC PHYSICIAN,

A MONTHLY JOURNAL OF

HOMŒOPATHIC MATERIA MEDICA AND CLINICAL MEDICINE.

"If our school ever gives up the strict inductive method of Hahnemann, we are lost, and deserve only to be mentioned as a caricature in the history of medicine."—CONSTANTINE HERING.

Vol. XIX. DECEMBER, 1899. No. 12.

### EDITORIAL.

DR. HERING'S REPERTORY OF TOOTH-ACHE.—Dr. Hering's admirable repertory of tooth-ache was first published in his *Domestic Physician* and afterward reproduced by his friend and admirer, Dr. Charles G. Raue, in his *Special Pathology and Therapeutic Hints.* By it the editor of THE HOMŒPATHIC PHYSICIAN has made many a seemingly miraculous cure of tooth-ache. Careful differentiation of the symptoms is absolutely necessary if we would achieve success in conquering this excessively painful complaint. This repertory of Dr. Hering more than anything else enables us to make this differentiation successfully and the editor can warmly testify to its usefulness in finding the simillimum. Many of the members of our school continually make use of this help, but there is a large number of the profession who seem to have forgotten this as well as many other valuable guides published in Dr. Raue's excellent book.

To once more remind the profession, and at the request of one or two members, we reproduce the repertory in this number of the journal; at the same time reminding our readers that it may be found combined with many other useful indications for treating tooth-ache in Dr. Raue's *Special Pathology and Therapeutic Hints*, third edition, page 246.

It has not been deemed advisable to reproduce all the notes there found, as the book itself is still in print and can be purchased of the publishers, Bœricke & Tafel.

